# A Novel Quantitative Spasticity Evaluation Method Based on Surface Electromyogram Signals and Adaptive Neuro Fuzzy Inference System

**DOI:** 10.3389/fnins.2020.00462

**Published:** 2020-05-25

**Authors:** Song Yu, Yan Chen, Qing Cai, Ke Ma, Haiqing Zheng, Longhan Xie

**Affiliations:** ^1^Shien-Ming Wu School of Intelligent Engineering, South China University of Technology, Guangzhou, China; ^2^Department of Rehabilitation Medicine, The Third Affiliated Hospital, Sun Yat-sen University, Guangzhou, China; ^3^School of Mechanical and Automotive Engineering, South China University of Technology, Guangzhou, China

**Keywords:** spasticity assessment, ANFIS, surface electromyogram, Modified Ashworth scale, stroke

## Abstract

Stroke patients often suffer from spasticity. Before treatment of spasticity, there are often practical demands for objective and quantitative assessment of muscle spasticity. However, the common quantitative spasticity assessment method, the tonic stretch reflex threshold (TSRT), is time-consuming and complicated to implement due to the requirement of multiple passive stretches. To evaluate spasticity conveniently, a novel spasticity evaluation method based on surface electromyogram (sEMG) signals and adaptive neuro fuzzy inference system (i.e., the sEMG-ANFIS method) was presented in this paper. Eleven stroke patients with spasticity and four healthy subjects were recruited to participate in the experiment. During the experiment, the Modified Ashworth scale (MAS) scores of each subject was obtained and sEMG signals from four elbow flexors or extensors were collected from several times (4–5) repetitions of passive stretching. Four time-domain features (root mean square, the zero-cross rate, the wavelength and a 4th-order autoregressive model coefficient) and one frequency-domain feature (the mean power frequency) were extracted from the collected sEMG signals to reflect the spasticity information. Using the ANFIS classifier, excellent regression performance was achieved [mean accuracy = 0.96, mean root-mean-square error (RMSE) = 0.13], outperforming the classical TSRT method (accuracy = 0.88, RMSE = 0.28). The results showed that the sEMG-ANFIS method not only has higher accuracy but also is convenient to implement by requiring fewer repetitions (4–5) of passive stretches. The sEMG-ANFIS method can help stroke patients develop proper rehabilitation training programs and can potentially be used to provide therapeutic feedback for some new spasticity interventions, such as shockwave therapy and repetitive transcranial magnetic stimulation.

## Introduction

Spasticity is a clinical symptom prevalent in stroke patients. In the common definition, spasticity is a motor dysfunction resulting from hyperexcitability of the stretch reflex, characterized by a velocity-dependent increase in resistance during passive stretches (Lance, 1980). The typical manifestation of upper extremity is flexor spasticity (Trompetto et al., 2014), which can cause pain and movement disorders, affecting the daily life quality of patients (Truini et al., 2013). There are a variety of methods for treating post-stroke spasticity, including non-pharmacological treatments [such as physical therapy (Gracies, 2001), orthoses (Basaran et al., 2014), and rehabilitation robotics (Crea et al., 2017)] and pharmacological treatments [such as oral treatments (Hulme et al., 1985) and injectable treatments (Patel, 2011)]. To design optimal spasticity treatment plans, it is important to evaluate spasticity accurately (Park et al., 2012). However, due to the complex and multifactorial nature of this phenomenon, which may involve nerve factors (central and peripheral) and non-neural factors (rheological properties of the muscle), quantifying spasticity remains a challenge and an unresolved problem (Stecco et al., 2014; Li and Francisco, 2015).

In the field of stroke rehabilitation, a commonly used evaluation method is the Modified Ashworth Scale (MAS). Based on the perceived resistance and the range of the elbow joint where resistance exists during passive stretching, the MAS classifies the degree of spasticity into six grades (0, 1, 1+, 2, 3, 4) (Bohannon and Smith, 1987). The MAS is widely used in the clinical field because of its simple operation. However, the MAS relies heavily on the subjective judgments of rehabilitation therapists and does not cohere with the velocity dependence of the spasticity definition (Lance, 1980). In addition, the semiquantitative descriptions in the MAS, such as “slight increase in muscle tone” (MAS 1, MAS 1+) and “more marked increase in muscle tone” (MAS 2), can easily lead to ambiguous results between “1 and 1+” and “1+ and 2” (Pandyan et al., 1999).

To overcome the subjective shortcoming of the MAS, Levin and Feldman proposed the tonic stretch reflection threshold (TSRT) method to quantify the degree of spasticity (Levin and Feldman, 1994). The TSRT evaluates the excitability of the motor neurons caused by both descending and segmental effects, and the measurement of these effects is the stretch reflex threshold (SRT, also called DSRT), which is the integral part of the lambda model of motor control (Feldman, 1986). The DSRT depends on the angle at which motoneurons and muscles start to be recruited (reflected in surface electromyogram (sEMG) signals) when the patient’s elbow joint is passively stretched at a given velocity (Levin et al., 2000). The TSRT can be obtained by linear fitting of a series of stretching velocities with DSRT values. The TSRT method is related to the resistance orientation through the range of motion in the MAS and quantifies the degree of spasticity from the objective joint angle. In addition, the TSRT is highly consistent with the definition of spasticity (Lance, 1980) and has been widely used to develop wearable devices for measuring spasticity in recent years (Kim et al., 2011; Germanotta et al., 2017).

However, in the TSRT method, a series of evaluation trials [such as 56 repetitions (Levin and Feldman, 1994), 30 repetitions (Marques et al., 2019), and 20 repetitions (Calota and Levin, 2009)] with different stretch velocities must be very carefully implemented in advance, which can easily lead to the boredom and fatigue of patients and therapists. Moreover, in a series of passive stretching processes, the spasticity of the muscles will decrease gradually (Watanabe, 2004). This decrease will lead to inconsistent MAS scores corresponding to the initial and final collected experimental data for one patient, resulting in the accuracy deviation of the TSRT method. These two points determine that the TSRT method still needs to be improved in clinical applications. To solve the above two issues of the TSRT method and evaluate spasticity more conveniently, we proposed an evaluation method based on the sEMG and adaptive neuro fuzzy inference system (i.e., the sEMG-ANFIS method).

sEMG signals can provide evidence of motor unit spontaneous discharges in stroke patients with spasticity and are thus widely used in the investigation of pathophysiology underlying spasticity (Li et al., 2006; Kallenberg and Hermens, 2011). Among stroke patients with spasticity, studies showed that there is an increase in the amplitude and frequency components of sEMG signals evoked from stretch reflex (Edgerton and Roy, 2010; Hu et al., 2018). It is suggested that the features in the time-domain and frequency-domain of sEMG signals can be utilized to evaluate the spasticity. Although sEMG signals were used to detect the SRT in TSRT method, the time-domain features and frequency-domain features of sEMG signals were not analyzed. In addition, time-domain features of root mean square (RMS) and frequency-domain features of mean power frequency (MPF) in sEMG signals have been used for MAS level classification (Zupan et al., 1998; Wang et al., 2017). This finding shows that time-domain and frequency-domain features of sEMG signals can be used for spasticity assessment. However, the time-domain and frequency-domain features of sEMG signals have not been used for the quantitative assessment of spasticity.

ANFIS is a kind of artificial neural network that has the advantages of the learning and adaptive abilities of a neural network and the reasoning ability of a fuzzy system (Jang, 1993). Thus, ANFIS is widely used to build regression models (Chang and Chang, 2006; Dariane and Azimi, 2016). In addition, Raj and Sivanandan (2017) proposed a combination of sEMG and ANFIS in elbow kinematics estimation, and the results showed that the ANFIS model was more accurate than the multi-layered perceptron (MLP) model. This finding shows that ANFIS is suitable to be combined with sEMG signals for the task of regression. Therefore, we chose ANFIS as a classifier.

In summary, the sEMG-ANFIS method was proposed to quantitatively evaluate the spasticity. Specifically, we collected sEMG signals from four elbow flexors or extensors (biceps brachii, triceps brachii, brachioradialis, and brachialis) of spasticity patients and used ANFIS for building the spasticity evaluation regression model. Only 4–5 repetitions passive stretches were required. Compared with the multiple stretches of the TSRT method, the implementation was greatly simplified. The results showed that the sEMG-ANFIS method also had higher accuracy than the TSRT method.

The remaining structure of this paper is as follows: Section Experimental Protocols introduces the experimental protocol, which includes participants, data acquisition devices and experimental setup. Section Materials and Methods is the methods, which includes active segment detection, window separation processing, feature extraction, feature reduction, the regression model and the evaluation function. Sections Results and Discussion provide experimental results and discussion, respectively. Finally, Section Conclusion summarizes this paper.

## Experimental Protocols

### Participants

In this paper, 15 people were recruited to participate in the experiment, including 11 stroke subjects and 4 healthy subjects. Before participating in the experiment, all subjects were informed of the experiment. This study was approved by the Ethics Board of the Medical School, South China University of Technology. All the research was performed in accordance with the Declaration of Helsinki.

All stroke patients were screened by two rehabilitation therapists. The inclusion criteria were as follows: (1) suffering from upper extremity flexor elbow muscle spasticity; (2) no joint contracture; and (3) voluntary participation in the experiment. Stroke patients were excluded if they had the following symptoms: (1) pain; (2) suffering from other central nervous system diseases that can cause spasticity, such as Parkinson’s syndrome or multiple sclerosis; (3) suffering from other diseases that may hinder upper limb motion. Ultimately, 11 patients participated in the experiment, as shown in Table 1. The specific information of the four healthy subjects is shown in Table 2.

**TABLE 1:** Details of the 11 stroke subjects.

Subject	Age	Brain lesion	Time since stroke (months)	spastic side	MAS grade
1	36–40	Brain infarction	21	Right	1+
2	66–70	Brain hemorrhage	3	Left	1+
3	36–40	Brain infarction	1	Right	2
4	56–60	Brain infarction	5	Left	2
5	56–60	Brain hemorrhage	3	Left	1
6	66–70	Brain infarction	1	Right	1
7	51–55	Brain hemorrhage	5	Right	1+
8	56–60	Brain infarction	6	Right	1
9	46–50	Brain hemorrhage	2	Left	1+
10	61–65	Brain infarction	8	Right	1+
11	66–70	Brainstem	3	Left	2

*To protect patients’ identity privacy, the age was reported as a 5 years range.*

**TABLE 2:** Details of the four healthy subjects.

Subject	Sex	Age	Tested side	MAS grade
1	M	23	Left	0
2	F	25	Right	0
3	M	26	Right	0
4	M	25	Right	0

## Data Acquisition

The sEMG data were recorded by a 16-channel wireless sEMG acquisition system (Ultium^TM^ Biomechanics system, Noraxon Ltd., United States) with a sampling rate of 2,000 Hz. The single sEMG sensor also has a built-in 9-axis inertial sensor (MPU9250) with a sampling rate of 400 Hz for recording three-axis gyroscope data. The gyroscope data were interpolated to match the length of sEMG data when all data was exported. In this study, sEMG data were combined with gyroscope data to implement the sEMG-ANFIS method and the TSRT method.

**FIGURE 1:**
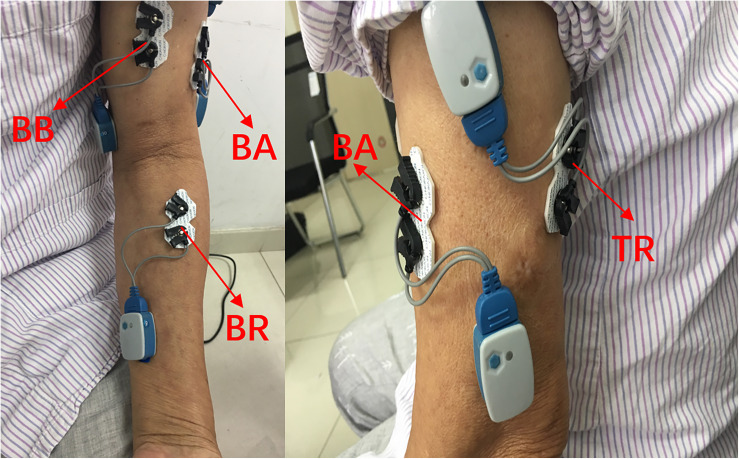
Electrode placement on the upper-limb muscles. BB, biceps brachialis; TB, triceps brachialis; BR, brachioradialis; BA, brachialis.

The collection of the sEMG signals was strictly in accordance with the recommended standards (Hermens et al., 1999; Konrad, 2005). In this paper, four elbow flexors or extensors related to elbow flexion and extension were selected: biceps brachii (BB), triceps brachii (TB), brachioradialis (BR), and brachialis (BA) (Andrews et al., 2012). After wiping with alcohol, four pairs of electrodes were applied to the corresponding muscle surfaces. The electrodes were placed as shown in Figure 1. The material of the surface electrode was AgCl, and the distance between the electrodes was 2 cm. The direction of the two electrodes was parallel to the muscle fibers. Then, the 1st–4th channels of the 16-channal sEMG acquisition instrument were used to collect the original sEMG signals. With an amplitude range of 100–5,000 μV and a frequency component of 0–500 Hz (Merletti et al., 1992), the sEMG signals were amplified 1,000 times and filtered through a 10–500 Hz filter in the wireless sEMG acquisition system.

### Experimental Setup

**FIGURE 2:**
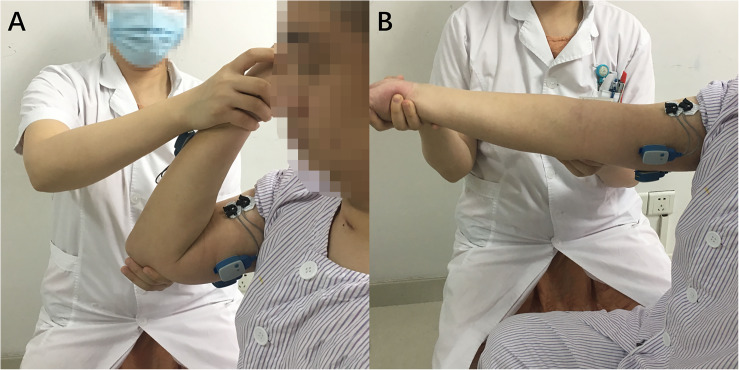
The start **(A)** and maximum **(B)** positions of spasticity assessment passive motion.

To minimize the diagnostic bias caused by the therapist’s subjective judgment, stroke patients were scored using MAS by two experienced rehabilitation therapists half an hour before the start of the experiment. The MAS score was the whole spasticity of patients’ flexors (i.e., biceps brachialis, brachioradialis, and brachialis). If the two MAS scores given separately were inconsistent, the therapists would exchange views and reach a single result. The final unified result was regarded as the patient’s true MAS score. We hypothesized that the unified results assessed by two therapists were objective. Then, one rehabilitation therapist held the elbow joint in one hand and passively stretched the affected arm with the other hand. In a single trial, the stretching start position was the maximum flexion position of the elbow joint. In the start position, as shown in Figure 2A, the rehabilitation therapist only helped the patients maintain the posture without applying any external force to the forearm. Next, the elbow joint was stretched to the maximum position that the upper arm and forearm could be extended, as shown in Figure 2B. Finally, the elbow joint was returned to the initial position, and the sEMG and gyroscope data were recorded. During the whole experiment, the forearm was in a neutral position, without any pronation or supination. The stretching speed was one of three different velocities, slow, normal and fast, which were distributed over 18–21 stretch experiments uniformly and were determined by the therapists subjectively. Between adjacent experiments, the patient rested for at least 10 s to avoid the effects of psychological or physical fatigue on the state of muscle tone.

## Materials and Methods

**FIGURE 3:**
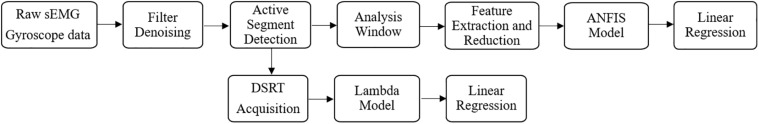
Flowchart of the procedure for sEMG processing.

**FIGURE 4:**
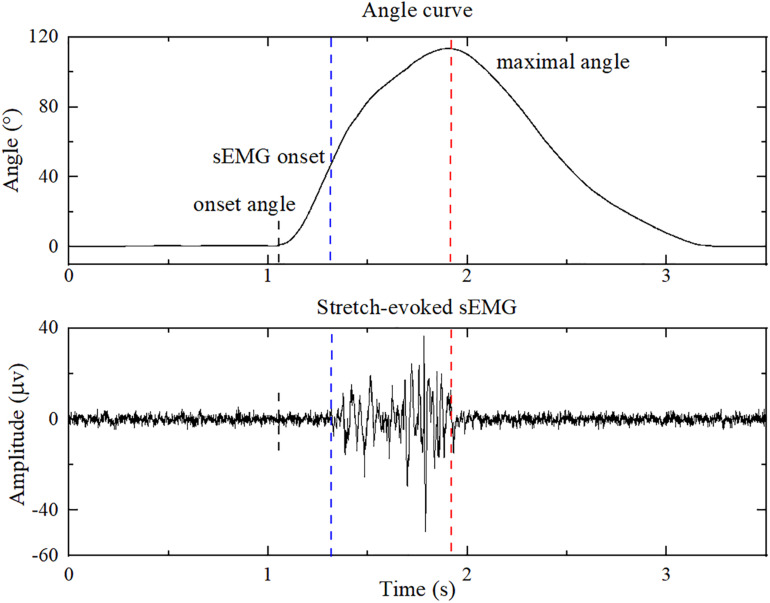
The sEMG of biceps brachialis and the corresponding elbow joint angle during a single passive stretching process. The blue dashed line shows the onset of sEMG evoked by passive stretch, and the red dashed line shows the maximal elbow joint angle. The onset angle was used for feature extraction of healthy subjects.

The data with fast stretching velocities were utilized in the sEMG-ANFIS method, and all data with three velocities were utilized in the TSRT method. All data were processed in MATLAB 2018b (The MathWorks Inc., Natick, United States), and the flowchart is shown in Figure 3. The sEMG signals were first subjected to a 20–350 Hz bandpass filter to remove ECG interference (Redfern et al., 1993), followed by a 50 Hz notch wave filter to remove power frequency interference. The three-axis gyroscope data of the brachioradialis were used to calculate the elbow joint angle curve and was first subjected to a 10 Hz low-pass filter to improve the signal-to-noise ratio. Then, the three-axis angle vectors were obtained by integrating the three-axis gyroscope signals, and the elbow joint angle was the superposition of the three-axis angles. The joint angle signal was corrected by the fact that the elbow joint angle in the start position is consistent with that in the end position. A representative elbow joint angle signal after correction is shown in Figure 4.

### Active Segment Detection

An improved threshold detection method based on empirical mode decomposition, the Hilbert envelope and the double threshold was employed to detect the corresponding onset of sEMG response of patients in each stretch trial (Silva et al., 2017). The onset and maximal elbow joint angles are marked as blue and red dashed lines in Figure 4. After autodetection of the onset and maximal elbow joint angle, feature vectors were extracted from the sEMG signals of the sEMG onset and maximal elbow joint angles. Since the stretch reflex threshold of healthy people is out of biomechanical range (Levin, 2016), the feature extraction progress of healthy subjects was from the start of the joint angle (onset angle) to the maximum joint angle. Double thresholds were set to detect the onset angle: the resultant angular velocity of three-axis angular velocities of 7.1°/s and the elbow joint angle of 1.2°, which were the peak value when therapists held the patients elbow in the static state in preliminary test, respectively. If the resultant angular velocity is greater than 7.1°/s, and the elbow joint angle is greater than 1.2°, the onset angle is detected.

### Analysis Window

Due to the randomness and non-stationarity of the sEMG signals, the analysis window, rather than the instantaneous value of the sEMG signals, was used (Smith et al., 2011). In the pretest, we used 32, 64, 128, and 256 ms windows to process sEMG signals and found that the 128 ms windows used in the ANFIS model had the highest regression accuracy under the same conditions. Therefore, an overlap analysis window with a length of 128 ms (256 data points) and a window sliding step size of 64 ms (128 data points) were used in this paper. Subsequent feature extraction and regression were based on these sliding windows.

### Feature Extraction

Thus far, features of the time-domain and frequency-domain have been widely used for sEMG signal processing (Phinyomark et al., 2018). In this paper, four common time-domain features and one common frequency-domain feature were selected to construct feature vectors. They were root mean square (RMS), variance (VAR), wavelength (WL) the 4th-order AR model coefficient (4th-ARMC) and the mean power frequency (MPF). Among these features, RMS and MPF have been used for the assessment of spasticity in SVM classifier (Wang et al., 2017) and ZCR has been used in the spasticity related research (Zadnia et al., 2018). In addition, WL and 4th-ARMC have also been used to combined with ANFIS for classification (Caesarendra et al., 2017). There were 20 feature vectors among the 4 channels in total. The respective features are described below:

(1) The RMS is the square root of the average power of the sEMG signals at a given analysis window. The formula is:
  (1) $${\text{RMS}}_{i}\left(t\right)=\sqrt{\frac{1}{M}\sum_{k=1}^{M}sEMG_{i}^{t}{\left(k\right)}^{2}}$$
  where *i* is the number of channels, t is the number of analysis windows, M is the number of all points in a window (M = 256), and k is the point currently in the analysis window.
(2) The ZCR is the number of times that the sEMG signal amplitude crosses the 0 axis; it can be formulated as:
  (2) $$\left\{ \begin{array}{c} sign\left(\text{x}\right)=\left\{ \begin{array}{c} 1x>0\\ 0else \end{array}\right.\\ ZCR_{i}\left(t\right)=\frac{1}{M-1}\sum_{k}^{M-1}sign\left(sEMG_{i}^{t}{\left(k\right)}^{*}sEMG_{i}^{t}\left(k+1\right)\right) \end{array}\right.$$
(3) The WL is the cumulative length of the sEMG signal over time. It can be calculated as follows:
  (3) $${\text{WL}}_{i}\left(t\right)=\sum_{k=1}^{M}\left|sEMG_{i}^{t}\left(k+1\right)-sEMG_{i}^{t}\left(k\right)\right|$$
(4) The AR model is a linear model used for time-series analysis of sEMG signals. It is defined as:
  (4) $$sEMG_{i}^{t}\left(k\right)=\sum_{j=1}^{q}a_{j}sEMG_{i}^{t}\left(k-j\right)+e_{i}^{t}\left(k\right)$$
(5) The MPF is the average frequency and can be calculated as the sum of the products of the EMG power spectrum and the frequencies divided by the total sum of the spectrum intensity (Oskoei and Hu, 2008). It can be formulated as follows:
  (5) $$MPF=\frac{\sum_{j=1}^{M}f_{j}P_{j}}{\sum_{j=1}^{M}P_{j}}\;$$
  where M is the length of the frequency bin, f_*j*_ is the frequency of the spectrum at frequency bin j and P_*j*_ is the sEMG power spectrum at frequency bin j.

### Feature Reduction

After feature extraction, principal component analysis (PCA) was used for dimensionality reduction. PCA transforms the raw data into a set of linearly independent representations of each dimension through linear transformation (Wold et al., 1987). It can be used to extract the main feature components of the data, and it is often used for dimensionality reduction of high-dimensional data. To minimize the loss of spasticity information, nine components whose variance contributions were more than 95% were used in this study. In other words, after the PCA dimension reduction, the feature matrix was reduced from the previous 20 dimensions to 9 dimensions.

### ANFIS Model

#### Adaptive Neuro Fuzzy Inference System (ANFIS)

The ANFIS model used in this paper, which combines a Sugeno system with a neural network, can predict the discrete MAS score exactly. For a general first-order Sugeno fuzzy model (Sugeno and Kang, 1988), the rules are as follows:

Rule 1: If x is A_1_ and y is B_1_, then
  (6) $$f_{1}=p_{1}x+q_{1}y+r_{1}$$
Rule 2: If x is A_2_ and y is B_2_, then
  (7) $$f_{2}=p_{2}x+q_{2}y+r_{2}$$

**FIGURE 5:**
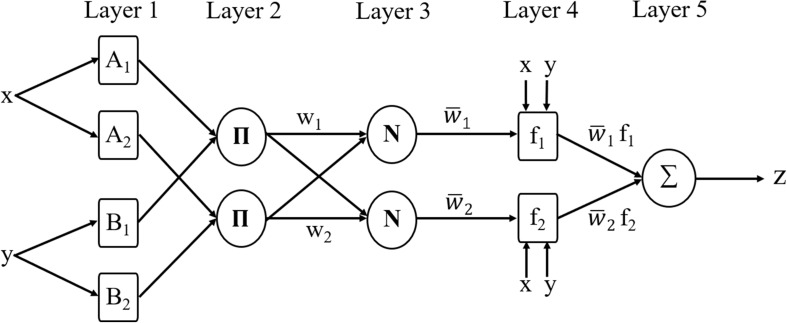
The framework of ANFIS.

The corresponding ANFIS architecture is as shown in Figure 5 (Jang, 1993). Nodes in the same layer have similar functions, and the functions of nodes in each layer can be described as follows:

Layer 1: every node is an adaptive node. This layer outputs the fuzzy membership grade of the inputs with the functions:

(8) $$O_{i}^{1}=\mu_{A_{i}}\left(x\right)i=1,2\;$$

(9) $$O_{i}^{1}=\mu_{B_{i-2}}\left(y\right)i=3,4$$

where x and y are the inputs of node i. A_i_ and B_i–__2_ generate a linguistic label when coupled with node i. The membership function for A can be any appropriate parameterized membership function, such as the Gaussian function:

(10) $$\mu_{A_{i}}\left(x\right)=e^{-\frac{1}{2}{\left(\frac{x-c}{\delta }\right)}^{2}}$$

where {*c*, δ} represents the parameter set. In this layer, the parameters are called premise parameters. If the values of this parameter set change, the shape of the Gaussian function will be changed accordingly. There are various possible membership functions for the fuzzy set A.

Layer 2: every node is a fixed node labeled with Π. The output of each node is the product of all the incoming signals, representing the firing strength of each fuzzy rule. The output can be represented as:

(11) $$O_{i}^{2}=w_{i}=\mu_{A_{i}}\left(x\right)\mu_{B_{i}}\left(y\right),i=1,2$$

Layer 3: every node is a fixed node labeled with N. The output of the ith node in this layer is the ratio of the ith rule’s firing strength to the sum of all rules’ firing strengths, indicating that it has a normalization role. The output in this layer can be calculated as:

(12) $$O_{i}^{3}={\bar{w}}_{i}=\frac{w_{i}}{w_{1}+w_{2}},i=1,2$$

Layer 4: all the nodes are adaptive nodes. In this layer, parameters are called consequent parameters. The output of each node in this layer is the product of the normalized firing strength from layer 3 and a first-order polynomial. Thus, the outputs in this layer can be calculated as:

(13) $$O_{i}^{4}={\bar{w}}_{i}f_{i}={\bar{w}}_{i}\left(p_{i}x+q_{i}y+r_{i}\right),i=1,2,\ldots$$

where ${\bar{w}}_{\text{i}}$ is the normalized firing strength from layer 3 and {*p*_i_, *q*_i_, *r*_i_} is the parameter set.

Layer 5: there is only one fixed node labeled with Σ in this layer. The output of this node is the summation of all incoming signals. Hence, the overall output of the model is given by:

(14) $$O_{i}^{5}=\sum_{i=1}^{2}{\bar{w}}_{i}f_{i}=\frac{\left(\sum_{i=1}^{2}w_{i}f_{i}\right)}{w_{1}+w_{2}}$$

#### Subtract Clustering

In this paper, the Sugeno-type FIS structure was generated using subtractive clustering. Subtractive clustering is a fast algorithm based on the density of data points in the feature space, and the amount of calculation required is linear with the data dimension. In this algorithm, the number and location of clusters in the data can be estimated automatically. Using subtractive clustering to generate fuzzy structure can greatly reduce the number of fuzzy rules and improve the robustness of structure with respect to noisy data (Chiu, 1994). In this algorithm, the points with the largest number of neighbors are selected as the cluster centers, and the candidates of the cluster centers are all points. Suppose that the samples used in training are n data points in m-dimensional space x = (x_1_, x_2_, …, x_*n*_), where xi = (x_i__1_, x_*i*__2_, …, x_im_) and i = 1,2, …, n. Considering that each data point is a potential cluster center, the density index D_*i*_ of data point xi can be defined as:

(15) $$D_{i}=\sum_{j=1}^{n}exp\left[-\frac{{||x_{i}-x_{j}||}^{2}}{{\left(\gamma_{a}/2\right)}^{2}}\right]$$

where the radius γ_a_ is a positive constant. Obviously, if a data point has multiple adjacent data points, that data point will have a higher density index value. γ_*a*_ defines a field of x_i_, and data points other than γ_a_ have little influence on the density index of the point. The data point with the largest density index is the first clustering center, which is set as x_c__1_, and the corresponding density index is Dc1. Next, we recalculate the density index performance of each data point according to the following formula:

(16) $$D_{i}=D_{i}-D_{c1}•exp\left[-\frac{{||x_{i}-x_{c1}||}^{2}}{{\left(\gamma_{b}/2\right)}^{2}}\right]$$

where γ_b_ = *k*γ_a_ is the neighborhood of cluster center xc1. γ_*b*_ defines a neighborhood to be reduced in density measurement to prevent a dense cluster center, typically γ_*b*_ = 1.5γ_*a*_. When *D*_i_ < 0, setting the density index of this data point to 0 will eliminate the possibility of this data point becoming the cluster center. After the density measures of all points are revised, the next cluster x_*c*__2_ is selected, and the density measures of all points are revised again. This process continues until the density index of the remaining data points is less than a certain threshold.

#### Parameters Setting

Next, the range of influence for the input variables was empirically specified as 0.65. The network structure had four rules and nine inputs, and each input had nine membership functions. The membership function was Gaussian function. The training epoch number was set as 20. The linear least-squares estimation was used to train the network.

In this paper, to evaluate the degree of spasticity in each patient using the sEMG-ANFIS method, the predicted value was calibrated by the uniform MAS scores given by two rehabilitation therapists. For convenience of calculation, the MAS scores 0, 1, 1+, and 2 were digitized as 0, 1, 1.5, and 2, respectively. A 15-fold leave-one-out cross-validation was applied to evaluate the performance of the sEMG-ANFIS method. The experimental data of 14 subjects were used as the training set, and the experimental data of the remaining subject were used as testing data. Since the analysis windows were taken as the minimal sample in this study, the average value of each window obtained from the sEMG-ANFIS method in the test set was taken as the last predicted score. After 15 rounds of circulation, each subject had a corresponding predicted MAS score.

The implementation of TSRT method was already proposed in a previous study (Zhang et al., 2019). In particular, the TSRT of healthy subjects was set to 140 degrees according to the biomechanical range (Levin, 2016). Similarly, to evaluate the ability of the TSRT method to assess the degree of spasticity, a 15-fold leave-one-out cross-validation was also implemented.

### Evaluation Function

For all subjects, evaluation scores were obtained using the sEMG-ANFIS method and the TSRT method. Both evaluation scores were calibrated using the uniform MAS scores. Therefore, the root-mean-square error (RMSE) and determination coefficient (R^2^) between the predicted score and the uniform MAS scores were used to evaluate the performance of the model. R^2^ and RMSE were formulated as follows:

(17) $$R^{2}=1-\frac{\sum_{i=1}^{N}{\left(y_{i}-{\hat{y}}_{i}\right)}^{2}}{\sum_{i=1}^{N}{\left(y_{i}-{\bar{y}}_{i}\right)}^{2}}\;$$

(18) $$\text{RMSE}=\sqrt{\frac{1}{N}\sum_{i=1}^{N}{\left(y_{i}-{\hat{y}}_{i}\right)}^{2}}\;$$

where N is the number of samples, *y*_i_ is the uniform MAS score, ${\bar{y}}_{\text{i}}$ is the mean value of uniform MAS scores and ${\hat{y}}_{\text{i}}$ is the predicted score.

To assess the subjectivity of the MAS, the Kappa statistic between the uniform MAS scores and the MAS scores given by the two therapists, the sEMG-ANFIS method and the TSRT method was used. In addition, to evaluate the robustness of the sEMG-ANFIS method, we randomly selected 1, 2, 3, 4, 5, and 6 data points of the fast passive stretching data from each subject to construct the 1-ANFIS model, 2-ANFIS model, 3-ANFIS model, 4-ANFIS model, 5-ANFIS model and 6-ANFIS model, respectively. To eliminate the contingency of the experimental results, we repeated the random selection process three times and compared the R^2^ and RMSE of these models. In the experimental process, due to operational errors and other reasons, only six data points of fast passive stretching data were obtained in some patients. Therefore, the process of establishing the 6-ANFIS model only occurred one time. Comparison between these models was achieved by a one-way analysis of variance (ANOVA) followed by a *post-hoc* Tukey test with a significance level of 0.05.

## Results

### Performance of the sEMG-ANFIS Method

**FIGURE 6:**
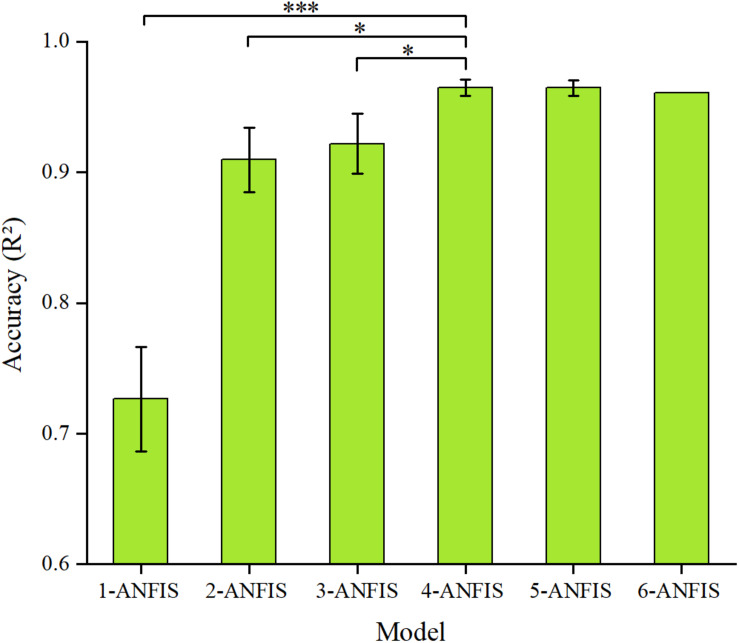
Comparison of the accuracy (R^2^ and RMSE) of the ANFIS model using different numbers of passive stretches. The result of the 4-ANFIS model has no significant differences with that of the 5-ANFIS model, but there is a significant difference with those of other models (^∗^0.01 < *p* ≤ 0.05; ^∗∗∗^*p* ≤ 0.001).

Figure 6 shows the prediction accuracy of the sEMG-ANFIS method with different passive stretching times in the form of mean ± SD. The results showed that the 4-ANFIS model and the 5-ANFIS model have higher accuracy than the other models, and there were no statistically significant differences between them. Then, the 4-ANFIS model (*R*^2^ = 0.96 ± 0.01, RMSE = 0.13 ± 0.01) was used as the representative model of the sEMG-ANFIS method for comparison with the TSRT method. There were also statistically significant differences between the 4-ANFIS model and the 1-ANFIS model, the 2-ANFIS model and the 3-ANFIS model. From 1-ANFIS model to 4-ANFIS model, the accuracy (R^2^) was significantly improved. After 4-ANFIS model, there was no significant change in accuracy (R^2^). From the above, it can be concluded that the sEMG-ANFIS method only requires several (4–5) passive stretches to establish a spasticity evaluation model with high accuracy and good robustness.

**FIGURE 7:**
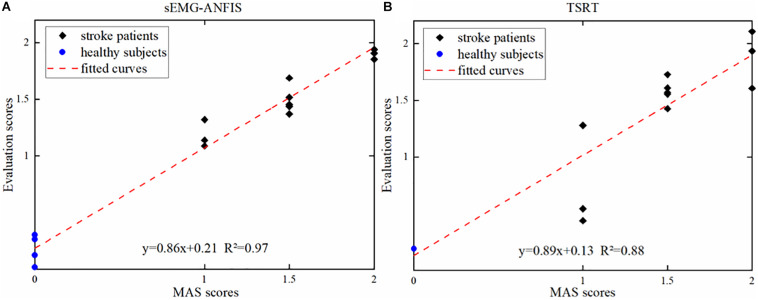
**(A)** The evaluation scores from the sEMG-ANFIS method and the uniform MAS scores. **(B)** The evaluation scores from the TSRT method and the uniform MAS scores.

Figure 7A shows one of the three results in the 4-ANFIS model with a scatter plot of the prediction scores and the uniform MAS scores. With an R^2^ between the predicted score and the uniform MAS scores of 0.97 and an RMSE of 0.12, the linear regression analysis showed the strong goodness of fit of the 4-ANFIS model. Figure 7B shows the evaluation scores obtained from the TSRT method. The TSRT method had a relatively large prediction error for patients with MAS grades 1 and 2. As a result, the RMSE between the TSRT method prediction scores and the corresponding MAS scores was 0.28, and the R^2^ was 0.88. It can be seen from Figures 6, 7 that the R^2^ of the 4-ANFIS model was higher than that of the TSRT method (0.97 > 0.88), and the RMSE was smaller than that of the TSRT method (0.12 < 0.28). Therefore, the conclusion that the sEMG-ANFIS method was better than the TSRT method in assessing the degree of spasticity was obtained from this study.

### The Subjectivity of MAS

**FIGURE 8:**
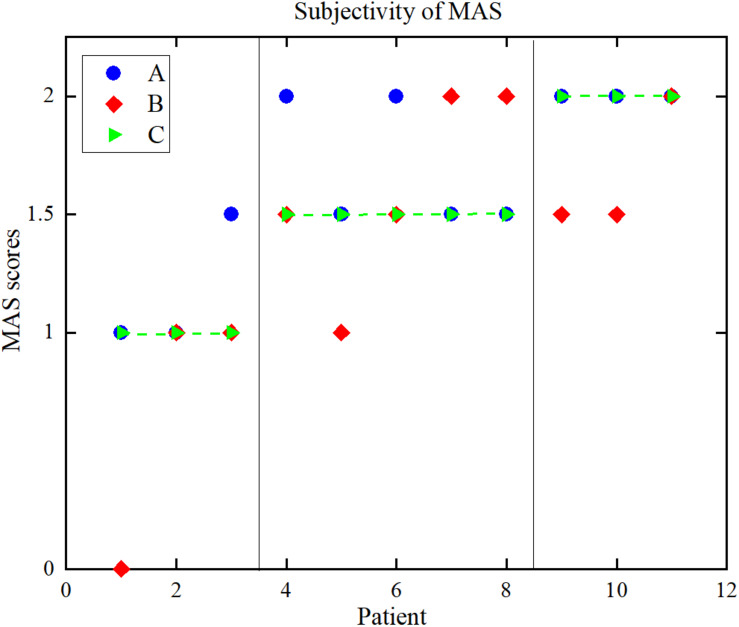
The distribution of the MAS scores. **(A)** Therapist A, **(B)** therapist B, **(C)** uniform results.

Figure 8 is a distribution map of the uniform MAS scores and the individual MAS scores given by pairs of therapists. The Kappa coefficients between the results given by the two rehabilitation therapists and uniform scores were 0.59 and 0.28. The Pearson correlation coefficients and Kappa coefficients between the uniform results and the results from the two therapists and two objective methods are shown in Table 3. For the sEMG-ANFIS method and the TSRT method, the prediction results were rounded to calculate the Kappa coefficient. From Table 3, the consistency of the diagnostic results between each of the two rehabilitation therapists and the uniform results were fair and moderate. The conclusion was drawn that the objectivity of MAS was poor. In comparison, the results of the sEMG-ANFIS method had the highest Kappa coefficient (0.86) and Pearson correlation coefficient (0.98), which indicates the objectivity of the sEMG-ANFIS method.

**TABLE 3:** Kappa coefficients, Pearson correlation coefficients between the scores of the two rehabilitation therapists, and the two methods, and the uniform scores.

Method	Kappa coefficient	Correlation coefficient
A and US	0.59	0.82
B and US	0.28	0.65
M1 and US	0.86	0.98
M2 and US	0.41	0.84

*A, therapist A; B, therapist B; US, uniform scores; M1, the sEMG-ANFIS method; M2, the TSRT method.*

## Discussion

This paper proposes a novel spasticity evaluation method that combines sEMG with ANFIS and compares it with the classical TSRT method. Considering the multiple passive stretches required in the TSRT method, the sEMG-ANFIS method, which only requires several (4–5) passive stretches, is convenient for operation. The RMS, WL, ZCR, 4th-ARMC, and MPF of the active segment sEMG signals were extracted to establish the ANFIS model for spasticity evaluation in this paper. From the comparison of R^2^ and RMSE, the sEMG-ANFIS method showed better accuracy than the TSRT method. Based on the sEMG-ANFIS method, a spasticity evaluation system can be established that does not rely on doctors or other professionals.

In this paper, a quantitative spasticity evaluation method that only needs several passive stretches was provided. Compared with the 56 (Levin and Feldman, 1994), 30 (Marques et al., 2019), or 20 (Calota and Levin, 2009) stretches of the TSRT method, our method is greatly improved in implementation simplicity. The main reason why the TSRT method requires many stretches lies in the unique definition of TSRT. TSRT refers to the angle at which motor neurons and muscles begin to be recruited at rest (i.e., at a velocity of zero). Under the actual experimental conditions, passive stretching could not be implemented at a velocity of zero. Therefore, the calculation of the TSRT value could only be obtained through a series of linear regressions of DSRT values at different stretch velocities (Levin et al., 2000). Due to the introduction of the intermediate variable DSRT, the TSRT method could not conveniently evaluate the degree of spasticity, and multiple passive stretches must be performed in advance. In contrast, our sEMG-ANFIS method maps directly from sEMG signals to a spasticity state without intermediate process conversion. The sEMG data for each group of passive stretches contains spasticity information, and we only need to decode the spasticity information from these sEMG data. Without an intermediate process, spasticity assessment could be performed several times for passive stretches. Moreover, since only a few passive stretches were required, the effect of the spasticity changes caused by multiple stretches on the assessment can be reduced.

In addition to the greatly improved simplicity, the accuracy of our method has also been improved. The mean R^2^ of the 4-ANFIS method reached as high as 0.96 ± 0.01 in this paper. Compared to the TSRT method, from the results (*R*^2^: 0.96 > 0.88, RSME: 0.13 < 0.28), the evaluation feasibility of the sEMG-ANFIS method was significantly improved. The performance of the sEMG-ANFIS method was also better than the method combined with the lambda model and the kinematic model (*R*^2^ = 0.93) in the previous study (Zhang et al., 2019). Compared to the previous study, the TSRT method in this paper had better performance. One reason for explaining the better performance of the TSRT method may lie in the fact that two therapists provided MAS scores and obtained more objective uniform MAS scores. In addition, compared to biomechanical method by measuring torque to evaluate spasticity (Wang et al., 2018), our method is portable and has more application prospects for wearable devices. Compared to recent proposed method using active movement to evaluate spasticity (Wang et al., 2019), our method has a wider audiences of spasticity patients for some of them suffer from muscle weakness.

In this paper, we also studied the subjectivity of MAS. Limited by the experimental conditions, we only found two rehabilitation therapists to evaluate the MAS levels. In this experiment, Therapist A’s assessment was closer to the uniform scores. There are many reasons for the low consistency between the results from the rehabilitation therapists and the uniform results. When interacting with different rehabilitation therapists, some patients may feel nervous. This could result in changes of muscle tone and affect the MAS assessment. In addition, different postures of the patient’s upper limb also change muscle tone (Watanabe, 2004), affecting the evaluation results of therapists.

In this study, continuous spasticity scores were output instead of several levels as in the MAS, which would play a positive role in the treatment of subsequent spasticity patients. In clinical situations, when therapists apply new methods, such as repetitive transcranial magnetic stimulation or shockwave therapy, to spasticity treatment (Wassermann, 1998; Wang, 2012), the MAS score cannot meet the requirement of reflecting therapy efficacy due to its subjectivity. Therefore, objective treatment feedback cannot be obtained during treatment. Our sEMG-ANFIS method has the potential to provide quantitative and objective feedback on spasticity treatment efficacy in the place of MAS and to promote related research on spasticity treatment. Our method can be packaged into easy-to-use operating software for the use of physiotherapists, which is also our future work. It is worthy to mention that since subtractive clustering was used to generate the ANFIS structure, our method was not relative time-consuming. After feature extraction, with a server configured with 48 GB graphics card and 128 GB of running memory, the mean processing delays of 1-2-3-4-5-6 ANFIS model were about 8, 10, 11, 15, 15, and 16 s, respectively. In addition, the study shows that sEMG signals can be used for compensation detection (Ma et al., 2019) and rehabilitation robot control (Koh et al., 2017). As the sEMG-ANFIS method was based on a 128 ms window, it can be combined with a compensation detection method to monitor the spasticity state and compensation pattern in real time and as feedback control in the application of a rehabilitation robot. Another application of the sEMG-ANFIS method is to help patients monitor their spasticity routinely when they are in the community.

There are still some defects in this study. First, the age of healthy subjects and patients were not matched, the healthy subjects were younger than patients. However, Zhang et al. (2019) has recruited age-mismatched participants to participant in the experiments for spasticity assessment and achieved good result, which shows that recruiting age-matched healthy subjects is not a crucial parameter that affects the experimental results. Then, there were no patients with MAS grades of 3 and 4 because of high stiffness or cognitive disability, which made it difficult for them to participate in the experiment. However, compared to the description of MAS grades 1–2 such as “slight increase in muscle tone” (MAS 1, MAS 1+) and “more marked increase in muscle tone” (MAS 2), the description of MAS grades 3–4 such as “considerable increase in muscle tone,” “passive movement difficult,” (MAS 3) and “affected part rigid” (MAS 4) are easier to distinguish (Pandyan et al., 1999). Therefore, our method can be combined with MAS to provide objective spasticity feedback for all grades of patients. Moreover, the feasibility and effectiveness of the regression framework based on sEMG and ANFIS signals were achieved offline. In our next study, we will achieve real-time output of the spasticity to promote the application of rehabilitation robots in the field of stroke rehabilitation.

## Conclusion

We developed a novel method based on sEMG and ANFIS for the convenient, objective and quantitative evaluation of spasticity. Four healthy subjects and 11 stroke patients with spasticity were recruited to participate in the experiment, and the sEMG signals from four elbow flexors or extensors were collected during fast passive stretching. Five time-domain or frequency-domain features were extracted from sEMG signals, and the ANFIS model was established. Our results showed the existence of a strong relationship between the MAS scores given by therapists and the predicted scores based on our methods (mean *R*^2^ = 0.96, mean RMSE = 0.13). Compared to the classic TSRT method (*R*^2^ = 0.88, RMSE = 0.28), the sEMG-ANFIS method is not only convenient for implementation but also has higher accuracy. This proves that our method can be used as a more convenient and quantitative method to replace traditional MAS and TSRT methods.

## Data Availability Statement

The datasets for this article are not publicly available because the datasets in this manuscript will be used for further research. Requests to access the datasets should be directed to LX, melhxie@scut.edu.cn.

## Ethics Statement

This study was carried out in accordance with the recommendations of the SCUT Research Ethics Guidelines and Researcher’s Handbook, Ethics Board of the Medical School, South China University of Technology, with written informed consent from all subjects. All subjects gave written informed consent in accordance with the Declaration of Helsinki. The protocol was approved by the Ethics Board of the Medical School, South China University of Technology.

## Author Contributions

SY designed the research and participated in the entire research including data collection, data processing, ANFIS-model construction, result interpretation, manuscript drafting and revisions. LX and YC designed the research and participated in the data collection and revisions of the manuscripts. QC, KM, and HZ participated in the data collection and analysis of the results.

## Conflict of Interest

The authors declare that the research was conducted in the absence of any commercial or financial relationships that could be construed as a potential conflict of interest.
